# Using the Ensuring Quality Information for Patients Tool to Assess Patient Information on Appendicitis Websites: Systematic Search and Evaluation

**DOI:** 10.2196/22618

**Published:** 2021-03-26

**Authors:** Shahi Ghani, Ka Siu Fan, Ka Hay Fan, Lorenzo Lenti, Dimitri Raptis

**Affiliations:** 1 St George's, University of London London United Kingdom; 2 Imperial College London London United Kingdom; 3 Department of HPB Surgery and Liver Transplantation Royal Free Hospital London United Kingdom

**Keywords:** appendicitis, patient information, EQIP tool, quality, tool, surgery, online health information, internet, health-seeking, behavior, review

## Abstract

**Background:**

Appendicitis is a common surgical problem among the young adult population, who are likely to use the internet to obtain medical information. This information may determine the health-seeking behavior of an individual and may delay medical attention. Little is known regarding the quality of patient information on appendicitis on the internet, as this has not been previously studied.

**Objective:**

The aim of our study was to identify the quality of information regarding appendicitis on websites intended for the public.

**Methods:**

We conducted a systematic review of information on appendicitis available online using the following 4 search terms in google: “appendicitis,” “appendix,” “appendectomy,” and “appendicectomy”. The top 100 websites of each search term were assessed using the validated Ensuring Quality Information for Patients (EQIP) tool (score 0-36).

**Results:**

A total of 119 websites met the eligibility criteria for evaluation. The overall median EQIP score for all websites was 20 (IQR 18-22). More than half the websites originated from the USA (65/119, 54.6%), and 45.4% (54/119) of all websites originated from hospitals, although 43% (23/54) of these did not mention qualitative risks from surgery. Incidence rates were only provided for complications and mortality in 12.6% (15/119) and 3.3% (4/119) of all websites, respectively.

**Conclusions:**

The assessment of the quality and readability of websites concerning appendicitis by the EQIP tool indicates that most sites online were of poor credibility, with minimal information regarding complication rates and mortality. To improve education and awareness of appendicitis, there is an immediate need for more informative and patient-centered websites that are more compatible with international quality standards.

## Introduction

In the modern era, the increasing accessibility and availability of information has promoted the internet as the primary source for patient information. The access to countless sources of information can cater to every need by providing jargon-free material for the wider public while making details available for those who seek in-depth knowledge. Thus, many patients search online for medical information on their symptoms prior to consulting medical professionals, many of whom subsequently self-diagnose based on these online sources [1,2]. Consequently, access to online medical information is critical to their decision-making process. However, with many sites containing potentially irrelevant or incorrect information, their credibility and reliability may present a barrier against seeking early medical help [3]. Furthermore, the use of unreliable websites may undermine patient relationships with health care professionals; at best, trust in healthcare may be affected, and at worst, presentations delayed by misinformed self-diagnoses may lead to poorer outcomes [4].

With a lifetime incidence of 8%, appendicitis is an affliction that is common enough to be familiar to the general public [5]. Hence, appendicitis, a leading cause of acute abdominal pain, and appendicectomy, its treatment, are likely to be terms that are searched by patients seeking further information. It has been shown that through providing education via high quality information on appendicitis and its risks, awareness and outcomes may be improved [6,7]. In order to make informed and competent health decisions, safe, reliable, and easily accessible information is essential; thus, the variable quality of internet patient resources warrant an evaluation of the quality and readability of this medical information.

The original Ensuring Quality Information for Patients (EQIP) tool is a checklist of 20 items used to assess written health care information [8]. Various aspects are considered, such as clarity of information, quality of written work, and website design. The EQIP tool has been used to evaluate information sources related to gallstone disease, transplant surgeries, eczema, liposuction, and, more recently, COVID-19 [9-13], demonstrating its applications across various disciplines and information types. We assessed the top-indexed websites related to appendicitis and appendicectomy using the modified EQIP tool for evaluation. The objective of our study was to evaluate the quality of information found on the top-searched websites that aim to provide patients with information on appendicitis.

## Methods

### Eligibility Criteria, Information Sources, and Data Selection

The most popular search engine, Google [14-16], was used to obtain a database of websites. Other search engines were not used in this study, as this would only lead to duplicate results. The searched terms were “appendix,” “appendicitis,” “appendicectomy”, and “appendectomy”. These were obtained using the Google AdWords Keyword Planner [17]. Formation of the database, analysis, and eligibility assessment of the websites were performed between September 2019 and February 2020. Previous work has suggested that patients limit their searches to well within the first 100 hits; therefore, the hits on each page were obtained until this target was reached [10]. The inclusion criterion was any website with information intended for patients. Websites were excluded if the literature was intended for scholars in scientific journals or if they were in a language other than English. Furthermore, links which directed individuals to purely video content or which were used for marketing purposes were also excluded. Upon exclusion, 119 websites were identified as eligible for analysis.

### Website Scraping

To obtain the URL database from the top 100 hits for each search engine, a website scraping tool was developed. This reduced the amount of time required for cutting and pasting links to the database. The custom Hypertext Preprocessor (The PHP Group) tool was designed to make HTTP requests to the search engines, mimicking the requests web browsers make when using a search engine. This tool made repeated requests, logging all of the hits per page with a target of 100 unique URLs. During this process, any duplicates were automatically removed within the individual search. If there were more hits on a page after the target of 100 websites was reached, these websites were also collected. The tool was run using a server based in Texas in the United States, although no preferences were chosen to limit searches to certain geographical areas.

### Data Entry

Each website was assessed independently by 4 assessors, SAG, KSF, KHF, and LL, all of whom are fluent in English. To assess each website, a Google Form containing the 36 EQIP items was used to evaluate criteria through yes, no, or “N/A” responses. Assessors also recorded the country of origin and the following source types: academic center, encyclopedia, health department, hospital, industry, news service, patient group, practitioner, professional society, or other. After the initial round of data entry, the websites were reassessed by another assessor, and any contradictory results were resolved by consensus.

### EQIP Tool

The original EQIP tool has been expanded to 36 criteria to provide a more robust and effective analysis of patient information. The modified EQIP tool sets out to satisfy the patient information collaboration guidelines of both the British Medical Association (BMA) [18] and the International Patient Decision Aids Standards (IPDAS) [19]. The modified EQIP tool consists of 36 items split into 3 domains: content (items 1-18), identification (19-24), and structure (items 25-36). Similar to previous studies, only yes or no options were provided for each item to avoid assessor subjectivity in partial answers. The option for “N/A” was also included if items were not relevant for the type of source. Websites which scored above the 75th percentile were deemed high-scoring websites.

### Additional Items Describing Mortality and Complication Rates of Surgery and Emergency Information

Questions were added to the Google Form to assess the variation in reported complication and mortality rates published by differing sources. The additional questions identified those websites that included rates for mortality and complications, and recorded the values given. Furthermore, a question was added to identify websites that included advice in the case of an emergency.

### Statistical Analysis

Continuous variables are reported as median and IQR and categorical variables as numbers and proportions in percentages. Continuous variables were compared with the Mann-Whitney and Kruskal-Wallis tests where appropriate. Proportions were compared with the Fisher exact test or chi-square test where appropriate. All *P* values were 2-sided and considered statistically significant when *P*<.05. A decision was made to dichotomize the EQIP score by using the 75th percentile as a cutoff point for discriminating high-scoring from low-scoring websites, as previously described and defined [9]. Statistical analysis was performed using R version 3.3.2 (The R Project for Statistical Computing, GNU General Public License version 2) and R Studio version 1.0.44 (RStudio) with the graphical user interface, rBiostatistics.com alpha version [20].

## Results

### Gathering of Websites With Information on Appendicitis and Its Management

**Figure 1:**
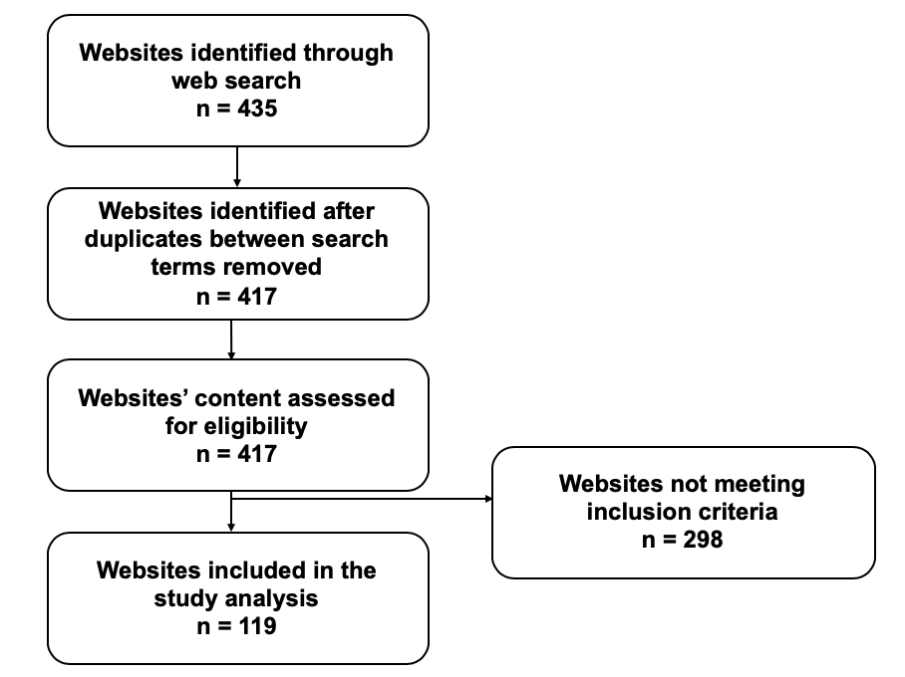
Workflow for identification of websites eligible for analysis.

To obtain a database of websites for analysis, the unique hits from each page using the search terms (“appendix,” “appendicitis,” “appendicectomy,” and “appendectomy”) were gathered. The workflow of this is shown in Figure 1. Although a target of 100 websites per term was used, additional hits on the last page of each search were gathered if they were unique, resulting in a total of 435 websites. Duplicate results obtained between search terms and websites failing to meet inclusion criteria were removed, resulting in 119 websites for analysis.

### Overall Quality of the Websites According to the Modified EQIP Tool

**Figure 2:**
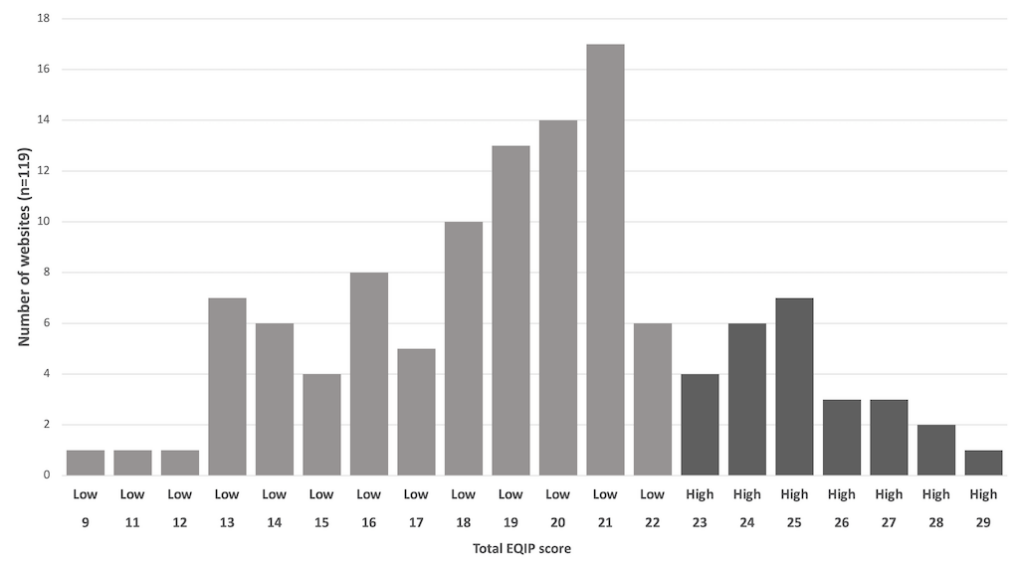
EQIP score of all websites assessed with patient information regarding appendicitis. High-scoring websites (EQIP score >75th percentile) are indicated by darker shadowing and labeling. EQIP: Ensuring Quality Information for Patients.

**Figure 3:**
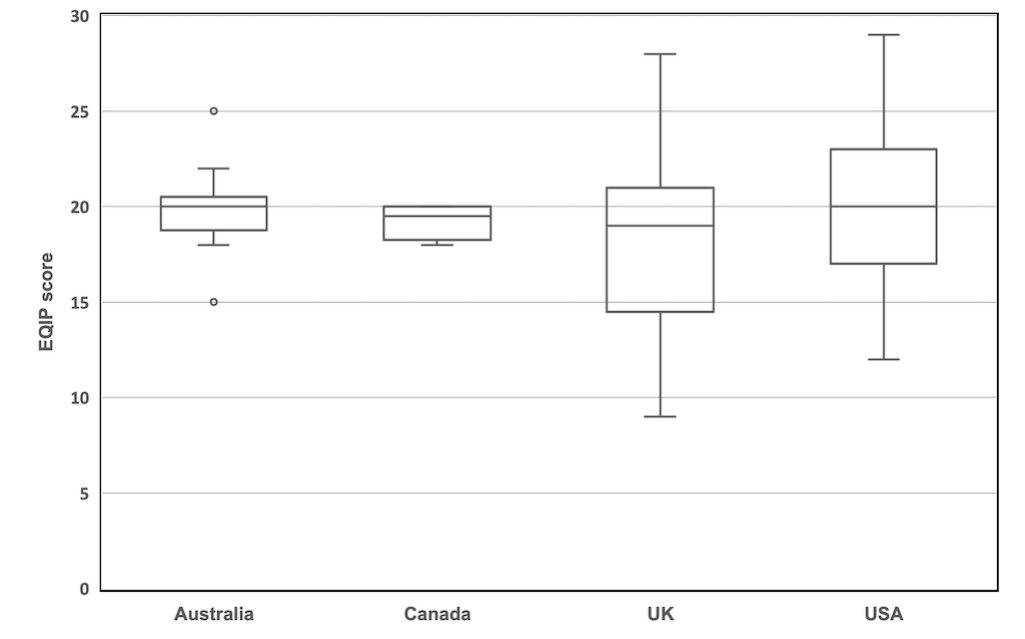
Distribution of EQIP scores per country of origin with more than one website. Boxplots represent median (within box) and IQR (lower and upper lines). EQIP: Ensuring Quality Information for Patients.

The distribution in EQIP score between all websites meeting inclusion criteria is shown in Figure 2. The country of origin and source of information for the database are shown in Table 1. Hospitals were the most common source of information, accounting for 45.4% (54/119) of the database, and 52% (28/54) of websites by hospitals originated from the United States. The country with the most websites was the United States, representing 54.6% (65/119) of the total, of which 26% (17/65) were high scoring. This represented 61% (16/26) of the total high-scoring websites. The distribution of EQIP scores by country of origin with more than one website is shown in Figure 3. Websites from the United Kingdom demonstrated the greatest variance in scores ranging from 9-28 with a median of 18 (Figure 3). Overall EQIP scores from countries with only 1 website were 24 for New Zealand, 19 for South Africa, 16 for Singapore, and 15 for India.

**Table 1:** Descriptive analysis of websites included in the study grouped by country of origin and source of information (N=119).

Parameters		Articles, n (%)
**Country**		
	Australia	10 (8.4)
	Canada	4 (3.4)
	India	1 (0.8)
	New Zealand	1 (0.8)
	Singapore	1 (0.8)
	South Africa	1 (0.8)
	United Kingdom	36 (30.3)
	United States	65 (54.6)
**Source of information**		
	Academic center	5 (4.2)
	Encyclopedia	6 (5.0)
	Industry	14 (11.8)
	Health department	12 (10.1)
	Hospital	54 (45.4)
	News service	19 (16.0)
	Patient group	1 (0.8)
	Professional society	8 (6.7)

 

Complication rates were included in 12.6% (15/119) of websites and varied between 0.2%-26%. Mortality rates were included in 3.3% (4/119) of websites and ranged between 0.001%-1.8%. Emergency advice was provided in 44.5% (53/119) of websites.

### EQIP Content Data

The items for the content domain of the EQIP tool are shown in Table 2. The median score achieved was 9 (50%), and the maximum score obtained was 16 (89%) of a possible 18 (Table 3). All of the high-scoring websites provided a description of the medical problem (item 3), definition of the purpose of intervention (item 4), and description of the qualitative risks and complications of appendicectomy (item 6; Table 2). High-scoring websites were found to describe how complications are handled (item 12) and provided details of other sources of reliable information (item 17) in 77% (20/26) and 73% (19/26) of cases, respectively; this was significantly less in the low-scoring websites (*P*<.001; Table 2). IQRs for the content domain are included in Table 3.

Among all websites (including low scoring), 95.7% (114/119) failed to address the costs and insurance issues related to appendicectomy (item 15). Furthermore, 97.4% (116/119) and 86.5% (103/119) of websites failed to describe the quantitative benefits (item 8) and risks (item 10) of appendicectomy, respectively (Table 2). Of note, 23 of the 54 hospital-published sources (43%) did not include any description of the qualitative risks and complications of surgery (item 9).

**Table 2:** Breakdown of results for the content domain (items 1-18) of the modified Ensuring Quality Information for Patients tool (N=119).

Content domain item	Websites scoring points for domain items, n (%)				OR^a^		95% CI		*P* value
	Overall (N=119)	High scoring (n=26)	Low scoring (n=93)						
1. Initial definition of which subjects will be covered	85 (71)	22 (85)	63 (68)	2.60		0.78-11.30		.14	
2. Coverage of the previously defined subjects (N/A^b^ if the answer is “no” for item 1)	90 (76)	23 (88)	67 (72)	N/A		N/A		.26	
3. Description of the medical problem/treatment/procedure	115 (97)	26 (100)	89 (96)	N/A		N/A		.58	
4. Definition of the purpose of the interventions	110 (92)	26 (100)	84 (90)	N/A		N/A		.20	
5. Description of treatment alternatives (conservative management)	45 (38)	14 (54)	31 (33)	2.32		0.88-6.22		.69	
6. Description of the sequence of the interventions and surgical procedure	96 (81)	25 (96)	71 (76)	7.66		1.12-331.81		.02	
7. Description of the qualitative benefits for the patient	90 (76)	25 (96)	65 (70)	10.63		1.58-456.75		.004	
8. Description of the quantitative benefits to the patient	3 (3)	2 (8)	1 (1)	7.49		0.38-455.58		.12	
9. Description of the qualitative risks and complications	76 (64)	26 (100)	50 (54)	N/A		N/A		<.001	
10. Description of the quantitative risks and complications	16 (13)	10 (38)	6 (6)	8.82		2.51-34.13		<.001	
11. Addressing quality-of-life issues	82 (69)	24 (92)	58 (62)	7.15		1.61-66.23		.003	
12. Description of how complications are handled	36 (30)	20 (77)	16 (17)	15.52		5.05-55.21		<.001	
13. Description of the precautions that the patient may take	37 (31)	13 (50)	24 (26)	2.85		1.06-7.75		.03	
14. Mention of warning signs that the patient may detect	80 (67)	24 (92)	56 (60)	7.82		1.76-72.15		.002	
15. Addressing medical intervention costs and insurance issues	5 (4)	1 (4)	4 (4)	0.89		0.02-9.55		.99	
16. Specific contact details for hospital services (N/A if not hospitals)	29 (24)	9 (35)	20 (22)	11.27		1.37-530.56		.01	
17. Specific details of other sources of reliable information/support	41 (35)	19 (73)	22 (24)	8.56		2.98 - 27.49		<.001	
18. Coverage of all relevant issues for the topic (summary item for all content criteria)	20 (17)	16 (62)	4 (4)	33.65		166.47-8.77		<.001	

^a^OR: odds ratio.

^b^N/A: not applicable.

**Table 3:** Analysis of EQIP scores obtained for each domain and overall.

Statistic	Content data^a^	Identification data^b^	Structure data^c^	Overall EQIP^d,e^
Median	9	3	8	20
Minimum	3	0	3	9
Maximum	16	6	10	29
Quartile 1	8	2	7	18
Quartile 3	11	4	9	22
IQR	3	2	2	4

^a^Total possible score for content=18.

^b^Total possible score for identification=6.

^c^Total possible score for structure=12.

^d^Total possible score=36.

^e^EQIP: Ensuring Quality Information for Patients.

### EQIP Identification

In the identification domain, the median score obtained was 3 (50%), and the maximum score obtained was 6 (100%; Table 3). Two websites, both of which were high scoring (overall score >75th percentile) obtained maximum points for this section. High-scoring websites were significantly better (58%, 15/26) than low-scoring websites (20%, 19/93) in providing a short bibliography of the evidence base for the information (*P*<.001; item 23; Table 4). IQRs for the identification domain are included in Table 3.

Furthermore, 97.4% (116/119) of all websites failed to include a statement about how patients were involved or consulted in the document’s production (item 24), and 68.0% (81/119) did not explicitly provide names of the persons or entities that financed the document (item 22; Table 4).

**Table 4:** Breakdown of results of the identification domain (items 19-24) and structure domain (items 25-36) of the modified Ensuring Quality Information For Patients tool (N=119).

Item		Websites scoring points for domain items, n (%)				OR^a^		95% CI		*P* value
		Overall (N=119)	High scoring (n=26)	Low scoring (n=93)						
**Identification domain**										
	19. Date of issue or revision	75 (63)	23 (88)	52 (56)	5.97		1.63-33.20		.002	
	20. Logo of the issuing body	117 (98)	26 (100)	91 (98)	N/A^b^		N/A		.99	
	21. Names of the persons or entities that produced the document	84 (71)	23 (88)	61 (66)	3.98		1.08-22.28		.03	
	22. Names of the persons or entities that financed the document	38 (32)	10 (38)	28 (30)	1.54		0.55-4.21		.35	
	23. Short bibliography of the evidence-based data used in the document	34 (29)	15 (58)	19 (20)	5.22		1.90-14.91		<.001	
	24. Statement about whether and how patients were involved/consulted in the document's production	3 (3)	2 (8)	1 (1)	7.49		0.38-55.58		.12	
**Structure domain**										
	25. Use of everyday language and explanation of complex words or jargon	107 (90)	24 (92)	83 (89)	1.44		0.28-14.42		.99	
	26. Use of generic names for all medications or products (N/A if no medications described)	55 (46)	20 (77)	35 (38)	1.14		0.06-70.72		.99	
	27. Use of short sentences (<15 words on average)	107 (90)	25 (96)	82 (88)	3.33		0.44-149.95		.46	
	28. Personal address to the reader	90 (76)	23 (88)	67 (72)	2.95		0.79-16.65		.12	
	29. Respectful tone	117 (98)	26 (100)	91 (98)	N/A		N/A		.99	
	30. Clear information (no ambiguities or contradictions)	117 (98)	26 (100)	91 (98)	N/A		N/A		.99	
	31. Balanced information on risks and benefits	64 (54)	24 (92)	40 (43)	15.59		3.53-143.74		<.001	
	32. Presentation of information in a logical order	116 (98)	26 (100)	90 (97)	N/A		N/A		.99	
	33. Satisfactory design and layout (excluding figures or graphs; see next item)	104 (87)	24 (92)	80 (86)	1.94		0.40-18.91		.52	
	34. Clear and relevant figures or graphs (N/A if absent)	34 (29)	10 (38)	24 (26)	1.85		0.30-20.62		.70	
	35. Inclusion of a named space for the reader’s notes or questions	12 (10)	2 (8)	10 (11)	0.69		0.07-3.59		.99	
	36. Inclusion of a printed consent form contrary to recommendations (N/A if not from hospitals)	1 (1)	1 (4)	0 (0)	N/A		N/A		.19	

^a^OR: odds ratio.

^b^N/A: not applicable.

### EQIP Structure

The median score obtained for the structure domain was 8 (66%), and the maximum score obtained was 10 (83%) of a possible 12 (Table 3). All high-scoring websites used a respectful tone (item 29), presented clear information (item 30), and delivered information in a logical order (item 32). High-scoring websites were found to include balanced information on risks and benefits (item 31) more frequently (24/26, 92%) than low-scoring websites (40/93, 43%; Table 4). In addition, 89.9% (107/119) of all websites failed to include a named space for readers’ questions, and 71.4% (85/119) failed to include clear and relevant figures or graphs (item 34; Table 4). IQRs for the structure domain are included in Table 3.

### Top 3 Websites According to the EQIP Tool

The websites scoring above the 99th percentile (EQIP score of 28) are shown in Table 5. The top-ranked resource, which scored 29 out of 36, was produced by the American College of Surgeons, displaying a comprehensive guide for patients to understand appendicitis and its management. Bupa health insurance and Medical News Today, each with a with an EQIP score of 28, were tied for the second ranking. All 3 sources provided information on symptoms, details of the procedure, preoperative and postoperative instructions, and guidance on complications.

**Table 5:** Websites scoring above the 99th percentile (EQIP score of 28).

Organization	Reference	Content data^a^	Identification data^b^	Structure data^c^	Overall EQIP^d,e^
American College of Surgeons	[21]	15	4	10	29
Medical News Today	[22]	16	4	8	28
Bupa	[23]	12	7	9	28

^a^Total possible score for content=18.

^b^Total possible score for identification=6.

^c^Total possible score for structure=12.

^d^Total possible score=36.

^e^EQIP: Ensuring Quality Information for Patients.

## Discussion

### Principal Findings

Based on our analysis, the quality of patient information regarding appendicectomy was shown to be of a moderate level, as reflected by the median overall score of 20 (IQR 18-22). Generally, websites tended to score well in the structure domain, reflected by the median score of 8 (IQR 7-9). This domain focuses on the ability of websites to display their information in a clear and logical manner. As technology is improving, it is becoming easier to produce websites and leaflets to a higher visual standard with minimal computer literacy. This presents a new challenge to patients with limited clinical understanding, as websites of poorer quality may appear similar to high quality sources of information. The increase in total EQIP score due to higher marks being obtained from the structure domain from the improvement of website quality is a phenomenon which has also been seen in regards to COVID-19 [13].

In the identification domain, high-scoring websites were better than low-scoring websites at providing a bibliography of the evidence base, potentially suggesting that these were written by individuals with experience in academia. It is therefore unsurprising that high-scoring websites more often included the names of the persons that produced the document. Contrastingly, the majority of websites (68.0%, 81/119) both high- and low-scoring, failed to include the names of the persons or entity that financed the document.

As appendicitis commonly affects younger individuals, it is possible that the decision to go to hospital is being made by a parent or guardian of the patient. This decision may be the result of a combination of factors, such as the severity of symptoms experienced by the patient, the health beliefs of the parent or guardian, and the quality of any information they may seek. Delays in presentation to hospital may increase the risk of complications, such as rupture; therefore, it is important that information regarding complications and warning signs is clearly described. Despite this clear need for quality, the scores achieved for the content domain were generally low. Only 67.2% (80/119) of websites mentioned warning signs to detect appendicitis, although 44.5% (53/119) of the websites did provide some form of advice in case of emergencies.

Hospitals contributed 45.4% (54/119) to the total database of websites, but 43% (23/54) of these failed to the specify qualitative risks of complications after surgery. It is also concerning that only 30.2% (36/119) of all websites provided a description of how complications may be handled. Over half of the websites (52%,28/54) from hospitals originated from the United States, possibly reflecting a degree of hesitancy by private hospitals to include information that may adversely affect customer decisions. This is further supported by the low number of websites reporting the incidence rate of complications. Although mortality rates are low for appendicitis, it is disappointing that this information was only provided by 4 websites, as this should be standard practice with the aim to fully inform patients.

To the best of our knowledge this is the only study to date to evaluate patient information regarding appendix surgery; therefore, it is not possible to evaluate how the EQIP tool compares to other scoring systems used for this condition. However, the EQIP tool has been described previously when evaluating the quality of information for gallstone disease (median EQIP score 15, IQR 13-18) [9], for clefts of the lip and palate (median EQIP score 19, IQR 16-22) [24], for bariatric surgery (median EQIP score 17, IQR 15-19) [25], for phalloplasty (median EQIP score 17.5, IQR 13-21) [26], for Dupuytren disease (median EQIP score 16, IQR 13-19) [27], for breast augmentation (median EQIP score 15, IQR 13-17) [28], donor information for living liver transplantation (median EQIP score 16, IQR 13-20) [10], and for COVID-19 (median EQIP score 18, IQR 15-20) [13]. The median score and IQR of this study are slightly higher than those in these studies. This may suggest that the general quality of information for appendicitis on the internet is higher than that of the previously studied diseases; however, this relatively high quality is likely due to the points gained in the structure domain in comparison to previous studies. The maximum score obtained in this study was 29, which was only achieved by 1 website and was still considerably lower than the maximum possible score of 36.

The first and second highest EQIP scores were achieved by websites from the United States and the United Kingdom, respectively. The United Kingdom also had the lowest-scoring website and lowest median among countries with more than one website. This indicates that the high-scoring websites in the United Kingdom are diluted by a majority of poor quality websites. The IQR range of scores for websites originating from Canada and Australia were much smaller, suggesting that, although there are fewer websites, they are generally of a higher quality.

### Limitations

There were a number of limitations for this study. Identification of search terms with Google AdWords Keyword Planner only provides commonly used search phrases by the wider public and may not be able to truly predict the search patterns of individuals seeking health information. Another limitation is that only websites in English language were evaluated; thus, the conclusions drawn might not be representative of patient websites in other languages. Furthermore, we have described the use of the EQIP in relation to appendicitis although the tool was not originally created for this specific purpose, and therefore this may be considered as a limitation. However, as the EQIP tool has previously been shown to be robust and effective for a number of surgical conditions [9,10,26,28], it is reasonable to expect this to also hold true for appendicitis. One may argue that there is no clear reason for why tools such as EQIP exist to evaluate patient information. The purpose of this tool is to enable a method of categorizing information so that we may learn what areas can be improved upon to produce patient information websites of higher quality. Our next step is to use the information gathered from this paper to design a website that will include information which has been found to be regularly omitted from previous work. Using solely the Google search engine introduced another limitation, as it is possible that the search engine had listed results not simply by popularity, but also by the geographical location of the requesting computer. Therefore, although geolocation features were disabled, the websites extracted could have still been centered around a particular location or continent, preventing a truly representative analysis of the top websites used globally. Finally, it is important to note that the findings of this study act as a snapshot of a particular point in time when the search was used; however, while search engine results do change over time, we consider the findings of this study to be representative of the information available to patients.

### Conclusions

In conclusion, the internet has become an essential source of information for our society. Our study showed that despite a growing body of web-based resources on appendicitis and appendicectomy, the currently available websites are generally of poor quality and inform patients inadequately. Although the clinicians responsible for each patient can provide patients with important, accurate, and relevant clinical information, the ability to direct patients to trustworthy internet resources may lead to better patient education and long-term outcomes. Online information is playing an increasingly significant role in patients’ attitude and can potentially affect willingness to accept and comply with medical advice [29]. With emergencies such as appendicitis, it is paramount to make emergency guidance widely available, especially when websites may be a patient’s first point of contact for information. Health care professionals should strive to educate patients on how to navigate and appraise internet-based resources in order to access the highest quality of information. Many studies have also identified similar problems and have not acted upon them, thus highlighting the urgent need to establish high quality websites and specific information evaluation tools to ensure the optimal patient education for a disease as common as appendicitis.

## Abbreviations

BMA: British Medical Association
EQIP: Ensuring Quality Information for Patients
IPDAS: International Patient Decision Aids Standards
